# The association of the PCSK9 rs562556 polymorphism with serum lipids level: a meta-analysis

**DOI:** 10.1186/s12944-019-1036-1

**Published:** 2019-04-30

**Authors:** Junlan Chuan, Zhengxu Qian, Yuan Zhang, Rongsheng Tong, Min Peng

**Affiliations:** 1 Personalized Drug Therapy Key Laboratory of Sichuan Province, Department of Pharmacy, Sichuan Academy of Medical Sciences & Sichuan Provincial People’s Hospital, School of Medicine, University of Electronic Science and Technology of China, Chengdu, 610072 China; 2 Department of Pharmacy, Sichuan Academy of Medical Sciences & Sichuan Provincial People’s Hospital, Chengdu, 610072 China; 3 Chengdu Institute of Biological Products Co., Ltd, Chengdu, 610000 China; 4 Department of Stomatology, Sichuan Academy of Medical Sciences and Sichuan Provincial People’s Hospital, School of Medicine, University of Electronic Science and Technology of China, Chengdu, 610072 China

**Keywords:** Proprotein convertase subtilisin/kexin type 9, Polymorphisms, Statin trearment, Meta-analysis

## Abstract

**Background:**

Studies had investigated the associations between proprotein convertase subtilisin/kexin type 9 SNP rs562556 and serum lipids levels and response to statin treatment, however, the results remained inconclusive. We conducted this meta-analysis to elucidate the relationship of rs562556 and serum lipids levels.

**Methods:**

All eligible studies met the inclusion criteria were retrieved from multiple databases. Relative data were extracted from each study. Review Manager (version 5.3.5) and STATA 12.0 software was used to perform this meta-analysis. Pooled standardized mean difference (SMD) with 95% CI was employed to evaluate the association of rs562556 with serum lipids levels.

**Results:**

A total of 7 eligible articles involving 4742 subjects were included in the final meta-analysis. The results revealed that the G carriers had lower levels of total cholesterol (SMD: 0.14, 95% Cl: 0.06–0.23, *P* = 0.001) and LDL-C(SMD: 0.13, 95% Cl: -0.55-0.22,*P* = 0.002) than the non-carriers. The statistical results also illustrated that the G carriers had lower relative risk (SMD: 1.38, 95% Cl: 1.02–1.85, *P* = 0.003) than the non-carriers.

**Conclusions:**

The results of the current meta-analysis for the first time indicated the relevance of rs562556 and lower serum cholesterol levels.

**Electronic supplementary material:**

The online version of this article (10.1186/s12944-019-1036-1) contains supplementary material, which is available to authorized users.

## Introduction

Proprotein convertase subtilisin/kexin type 9 (PCSK9), the ninth member of the subtilisin family of kexin-like proconvertases, was formerly known as neural apoptosis regulated convertase 1 (NARC-1) which was responsible for the cleavage of a variety of precursor proteins including neuropeptides, prohormones, cytokines, growth factors and other cell surface proteins [1, 2]. PCSK9 was also identified as the third gene implicated in autosomal-dominant hypercholesterolemia after low-density lipoprotein receptor (LDLR) and apolipoprotein B(APOB) [3, 4].

The pre-processed PCSK9 comprised four domains: the signal peptide (residues 1–30), the N-terminal pro-domain (residues 31–152), the catalytic domain (residues 153–425) and the C-terminal cysteine histidine rich domain (residues 526–692) [5]. PCSK9 is mainly expressed in liver, kidney and intestine [2]. It was secreted into circulation after auto-catalytically cleaving by the N-terminal prodomain [6–8]. Residues 61–70 in the N-terminal pro-domain were reported to be critical for self-cleavage, secretion, or LDLR-degrading activity [5]. Through the catalytic domain, PCSK9 effectively bound to the epidermal growth factor-like repeat A domain of LDLR and then rerouted LDLR to the lysosome for degradation. The C-terminal domain also mediated protein-protein interactions and influenced secretion and LDLR-reducing activity [5, 8]. PCSK9 prevented LDLR recycling to the cell membrane, reducing the endocytosis of low-density lipoprotein cholesterol (LDL-C) thus increasing he serum lipid levels.

PCSK9 is a highly polymorphic gene. Variants in the PCSK9 gene have been associated with variability of serum lipids levels especially the level of LDL-C [9, 10]. Gain-of-function mutations interferes with the recycling of LDLR to cell surface, which reduced the uptakes of LDL-C and increased LDL-C level [11]. Loss-of function decelerated the degradation of LDLR, increasing the number of LDLR on the surface and promoting circulating LDL-C absorbing into cells [12].Statins competitively inhibited HMG-CoA reductase activity, lowered cellular cholesterol concentrations and increased synthesis of LDLR as compensation [13–15]. Thus, loss of function mutations in the PCSK9 were expected to have a better response to statins suggesting that lipid-lowering by PCSK9 inhibitors may be synergistic to that achieved by statins treatment [16]. The LDL-C lowering effect may be counteracted by gain-of-function mutations in PCSK9 since they favored LDLR destruction [17, 18].

The PCSK9 rs562556 (c.1420G > A, I474V) variant in exon9, located within the linker domain between catalytic domain and C-terminal domain, was reported as a gain-of-function mutation [17]. Numerous studies in different ethnic group have been performed to explore the link between rs562556 and plasma LDL cholesterol levels [19]. Some of them further investigated the association of rs562556 and the effect of station treatment. However, the results were inconsistent and controversial and no meta-analysis has yet been conducted on it. Therefore, we conducted the current meta-analysis of all eligible studies to assess the associations of rs562556 variation with lipid traits.

## Material and methods

### Search strategy

All studies focused on the associations of PCSK9 rs562556 with plasma lipids level and the effect of statin treatment were identified. A systematic search was performed in Pubmed, Elsevier and Web of Science up to December, 2018 without restrictions. Different combinations of the following terms were applied: “PCSK9”, “proprotein convertase subtilisin/kexin type 9”, “neural apoptosis regulated convertase”, “NARC-1”, “rs562556”, “polymorphism”, “variant” and “mutation”. Two authors (JC, ZQ) conducted the research independently. We also performed a full manual search from the references of relevant articles for studies potentially missed in the primary searches.

### Study selection

This meta-analysis aimed to investigate the relationship between rs562556 and serum lipids levelS including total triglycerides (TG), total cholesterol (TC), LDL-C, and high-density lipoprotein cholesterol (HDL-C) before and/or after statin treatment. Studies were included in the meta-analysis when mean plasma lipid levels and standard deviations or standard errors by genotype were available. Reviews, case reports, pedigree-based studies, animal studies and reports with incomplete date were excluded.

### Data extraction

The unqualified and repeated studies were excluded after reviewed independently by two authors (JC,ZQ). Disagreements were consulted by the third person (MP). The following information was extracted from each included study: first author, year of publication, origin country, ethnicity, sample size, genotyping method, mean lipids level and standard deviations before and/or after statin treatment.

### Statistical methods

Due to the low frequencies of the GG genotype, more than half of the included studies reported the serum lipids level by pooling the GG and AG genotype. So a dominant model [(AG + GG) versus AA] was employed for calculation. Distributions of continuous variables were presented as mean ± standard deviation. We recalculated the mean ± standard deviation under dominant model when the candidate study did not report in this way. The strength of association was assessed and interpreted as pooled standardized mean difference (SMD) and its corresponding 95% confidence interval (CI). A *P* value < 0.05 was regarded as a statistically significant difference. Heterogeneity between studies was assessed using the chi-square-based “*Q*” test and inconsistency value (*I*^*2*^); *P* ≤ 0.05 and/or *I*^*2*^ > 50% indicated a significant heterogeneity. When *P* ≥ 0.05 and/or I^2^ < 50%, the Inverse-Variance fixed-effect model was used for the combined data, otherwise Inverse-Variance random-effects model was applied. Sensitivity analysis was performed by omitting each individual study in turn and performing all calculations using the remaining studies to assess the stability of the pooled results. Publication bias was assessed using Begg’s rank correlation test which was shown as a funnel plot (*P* < 0.05 was considered to be statistically significant). Funnel plot asymmetry was also examined by using Egger’s linear regression test. All statistical analyses were conducted with STATA 12.0 software (StataCorp, College Station, TX) and Review Manager (version 5.3.5) (Cochrane Collaboration, Oxford, United Kingdom).

## Results

### Study selection and baseline characteristics

**Fig. 1:**
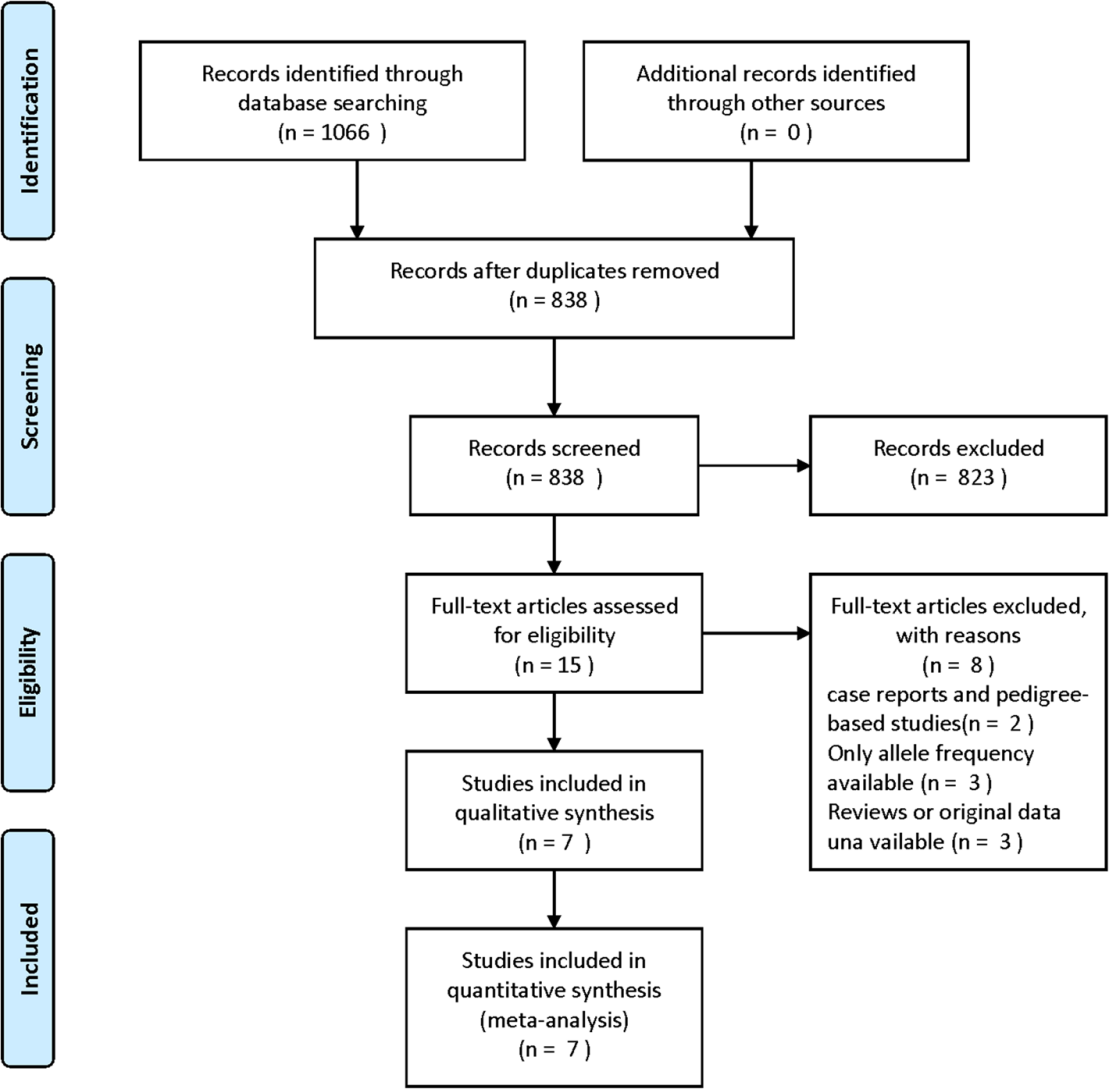
Flow diagram of study selection process for PCSK9 rs562556

PRISMA Flow Diagram documenting the details of study selection process is illustrated in Fig. 1. A total of 1066 potentially relevant articles were retrieved at the initial database search stage. Among them, only 15 articles were with respect to rs562556 polymorphisms [17, 19–32]. After applying inclusion and exclusion criteria, 7 eligible articles published between 2004 and 2017 were included in the final meta-analysis model [19, 22, 23, 26, 27, 30, 32]. The main features of all the included studies were summarized in Table 1. There are 4 studies performing genotyping using TaqMan assay, two studies using polymerase chain reaction-restriction fragment length polymorphism and one with exonic sequencing. Three of these studies involved Asian populations conducted in Thailand and Japan, one of the 7 studies was about Caucasian populations from Italy and another one researched on both Caucasian populations and African Canadians in Canada, the other two studies was based on Brazilian.

**Table 1:** Main characteristics of eligible studies included this meta-analysis

First Author	Year	Country	Ethnicity	Type of study	Sample size	Genotyping method	Number by Genotype	
							AA	AG/GG
Xavier	2017	Brazil	Brazilian	Cohort	182	TaqMan	122	60
Jeenduang	2015	Thailand	Thai population	Cohort	495	PCR–RFLP	471	24
Mayne	2013	Canada	Caucasian and African Canadians	Cohort	207	exonic sequencing	150	57
Norata	2010	Italy	Caucasian	Cohort	1541	Taqman	1051	490
Anderson	2014	Brazil	Brazilian	Cohort	299	TaqMan	191	108
Wanmasae	2016	Thailand	Thai population	Cohort	225	PCR-RFLP	217	8
Shioji	2004	Japan	Japanese	Cohort	1793	TaqMan	1704	89

### Heterogeneity and sensitivity analysis

If all of the 7 studies were included for statistical analysis, significance of heterogeneity was observed and the value of I^2^ = 100%. The sensitivity analysis was conducted via sequential analysis after omitting one study at a time to assess the effects of individual studies on the overall meta-analysis estimate. Sensitivity analysis suggested that the study Shioji 2004 obviously affected the overall Std Mean Difference values of correlations. Heterogeneity was specifically decreased (I^2^ ≤ 41.3%), when the study by Shioji 2004 was removed. So the reference study Shioji 2004 was excluded when assessing the association of PCSK9 rs562556 with serum lipid levels.

### Association between *PCSK9* rs562556 and plasma lipid levels

**Fig. 2:**
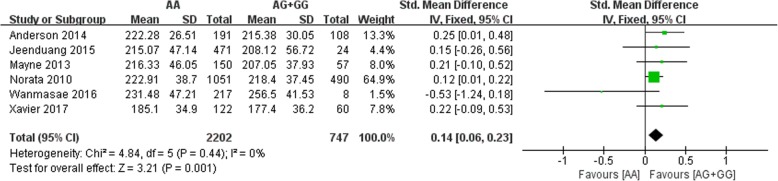
Forest plot of the association between PCSK9 rs562556 polymorphism and the serum levels of TC

**Fig. 3:**
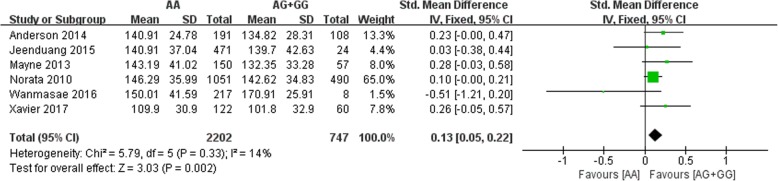
Forest plot of the association between PCSK9 rs562556 polymorphism and the serum levels of LDL-C

**Fig. 4:**
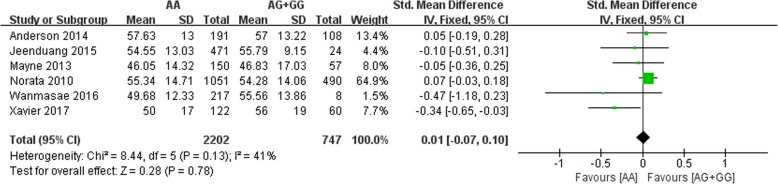
Forest plot of the association between PCSK9 rs562556 polymorphism and the serum levels of HDL-C

**Fig. 5:**
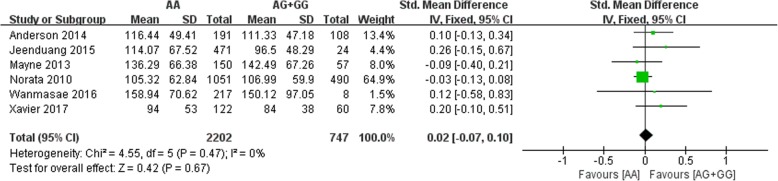
Forest plot of the association between PCSK9 rs562556 polymorphism and the serum levels of TG

All six included studies investigated the correlation between the rs562556 SNP and serum lipid levels. The results of our meta-analysis revealed that the G carriers had lower levels of TC (SMD: 0.14, 95% Cl: 0.06–0.23, *P* = 0.001) and LDL-C(SMD: 0.13, 95% Cl: 0.05–0.22, *P* = 0.002) than the non-carriers (Figs. 2, 3). No statistically significant difference in the levels of HDL-C(SMD: -0.01, 95% Cl: -0.07-0.10,*P* = 0.78) and TG(SMD: 0.02, 95% Cl: -0.07-0.10,*P* = 0.67) was detected between the G carriers and the non-carriers (Figs. 4, 5).

### Association between *PCSK9* rs562556 and relative risk

**Fig. 6:**
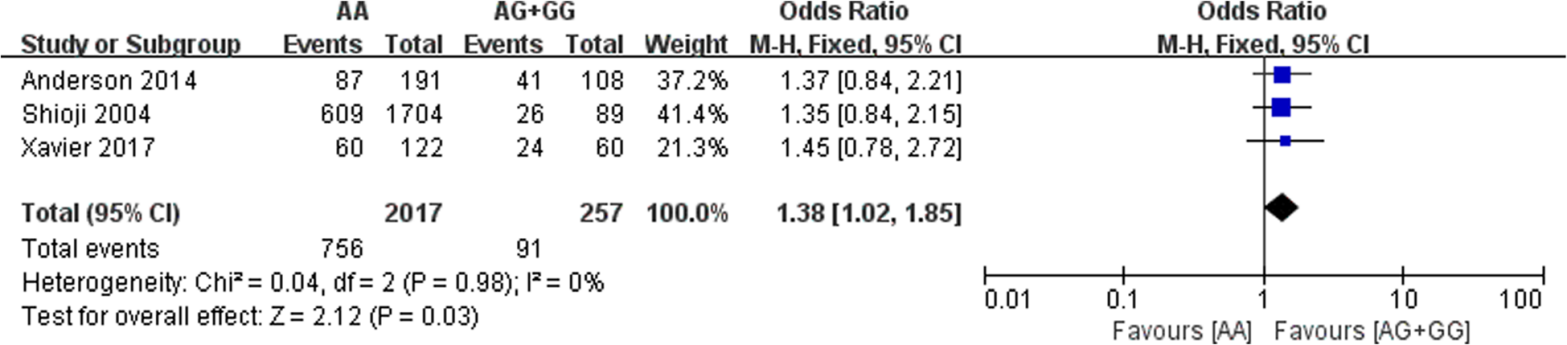
Forest plot of the association between PCSK9 rs562556 polymorphism and relative risk

Only three of the included studies investigated the associations between the rs562556 polymorphism and relative risk. One of them evaluated the PCSK9 polymorphisms rs562556 with serum lipids level in Polycystic Ovary Syndrome subjects (Xavier 2017). Another study investigated the influence of PCSK9 variants on plasma lipid profile on hypercholesterolemic and normolipidemic individuals (Anderson 2014). While the third study included myocardial infarction group and controls to assess the association between this polymorphism and the incidence of myocardial infarction (Shioji 2004). A total of 2274 with subjects were involved in the three studies. The statistical results also illustrated that the G carriers had lower relative risk (SMD: 1.38, 95% Cl: 1.02–1.85, *P* = 0.003) than the non-carriers (Fig. 6).

### Publication Bias analysis

**Fig. 7:**
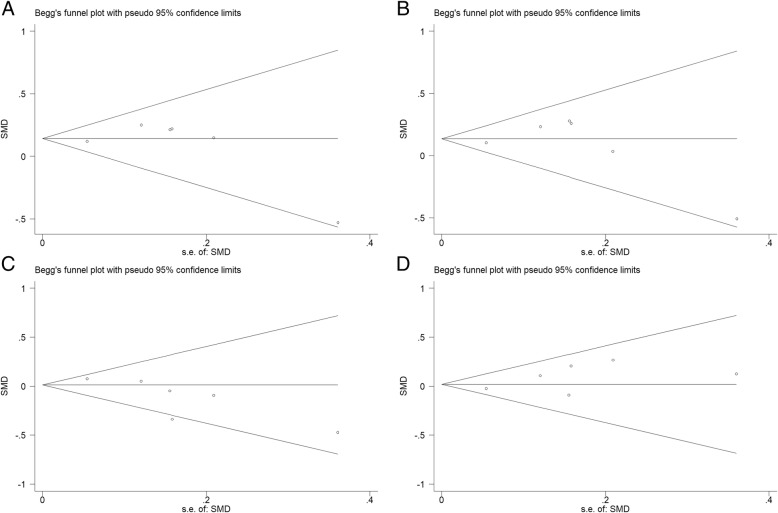
The funnel plot of publication bias detection between PCSK9 rs562556 polymorphism and the serum lipid levels (**a**: TC; **b**: LDL-C; **c**: HDL-C; **d**: TG)

Publication bias of the literature was assessed by visual inspection of the Begg’s funnel plots for asymmetry. Begg’s rank correlation test and Egger’s linear regression test were used to provide statistical evidence for funnel plot symmetry. The shapes of the funnel plots did not reveal any evidence of obvious asymmetry (Fig. 7 a, b, d), except a slight asymmetry in the case of analysis of rs562556 polymorphisms with the HDL-C levels (Fig. 7c). The Egger’s test gave the same result and all values of *P* > 0.05 (Table 2).

**Table 2:** Meta-Analysis results of PCSK9 rs562556 polymorphism and the serum lipid levels under dominant model (AG + GG versus AA)

Plasma Lipid	Analysis Method	Heterogeneity		SMD				Publication Bias	
		*I*^*2*^(%)	*P* value	Overall	Lower	Upper	*P* value	Begg	Egger
TC	fixed	0.0	0.44	0.14	0.06	0.23	0.001	0.452	0.751
LDL-C	fixed	14.0	0.33	0.13	0.05	0.22	0.002	0.452	0.852
HDL-C	fixed	41.0	0.13	0.01	-0.07	0.10	0.78	0.060	0.054
TG	fixed	0.0	0.47	0.02	-0.07	0.10	0.67	0.452	0.168

## Discussion

The main function of PCSK9 was binding the LDLR and promoting LDLR degradation in lysosome. It interfaced the recycling of LDLR to cell surface thus regulated the serum LDL-C level. Statins inhibited the cellular biosynthesis of cholesterol by competitive inhibition on HMG-CoA reductase, leading LDLR increasing as a feedback. A growing number of researches revealed that the polymorphisms of PCSK9 not only associated with plasma lipid levels but also affected statin treatment. Two of the most extensively studied variants of PCSK9 were rs505151 (c.2009G > A, E670G) and rs11591147 (c.137G > T, R46L). SNP rs505151 (c.2009G > A, E670G), a common gain-of-function mutation, was accompanied with higher TC and LDL-C and interacted with statin treatment. Variant allele carriers of E670G had no significant benefit from statin treatment compared to homozygous wild type carriers who did benefit [33, 34]. SNP rs11591147 (c.137G > T, R46L) was a rare loss-of-function mutation and associated with lower LDL-C concentrations [35]. But its effect on the statin treatment was inconsistent. Some studies found no significant differences in LDL-C reduction influenced by the genetic status and thus found no increased response to statin therapy in the R46L variant allele carriers [36]. But other studies reported that rs11591147 was significantly associated with LDL-C response to statin treatment [16]. Several other SNPs of PCSK9 like rs11206510, rs11583680 (c.158C > T, A53V) and rs562556 (c.1420G > A, I474V) was also reported to link with plasma lipids levels.

Since no meta-analysis has yet been conducted on rs562556 (c.1420G > A, I474V), our primary purpose was to assess the role of rs562556 on the serum lipids levels. To our knowledge, this was the first meta-analysis to comprehensively investigate the association between rs562556 and serum lipids levels. We did this meta-analysis and found that this variant were positively associated with lower levels of TC and LDL-C under dominant model. Subgroup analysis based on ethnicity suggested that rs562556 had correlation with lower levels of TC and LDL-C in Caucasian, African Canadian, Brazilian but not in Thai population. A study based on Japanese revealed that rs562556 had strong correlation not only with lower levels of TC, LDL-C and TG but also with higher levels of HLD-C (data not shown). This difference may be attributed to different regional influences, ethnicities, and lifestyles. No significant differences in the response to statin treatment were observed between G-carriers and non G-carriers. Only two studies were included to assess the response to statin treatment, this result should be treated prudently. More related studies were needed to verify this conclusion and to give more robust evidence.

Several unavoidable limitations of the present study should be noted. Firstly, the number of published studies and sample size were relatively small. A total of 2949 objects were included in the final analysis. Secondly, the objects including healthy population, hypercholesterolemics patients, polycystic ovary syndrome patients, varied widely considering their disease characteristics, which may weaken the statistic power of the findings of this meta-analysis. Thirdly, some of the included studies were case-control designed while the others were not. We recalculated and combined the data together, which may introduce inevitable interference for the final results.

## Conclusions

The current meta-analysis provided evidence of a positive association between rs562556 and lower TC and LDL-C levels.

## Additional file

Additional file 1: The funnel plot of publication bias detection between PCSK9 rs562556 polymorphism and the serum lipid levels (circle: TC; diamond: LDL-C; square: HDL-C; triangle: TG). (PNG 9 kb)

## Abbreviations

Cl: Confidence interval
HDL-C: High-density lipoprotein cholesterol
LDL-C: Low-density lipoprotein cholesterol
LDLR: Low-density lipoprotein receptor
NARC-1: Neural apoptosis regulated convertase 1
PCSK9: Proprotein convertase subtilisin/kexin type 9
SMD: Standardized mean difference
TC: Total cholesterol
TG: Total triglycerides
