# Supplementary figures and images for: The association of the PCSK9 rs562556 polymorphism with serum lipids level: a meta-analysis
Source: Lipids Health Dis. 2019 Apr 30;18:105. doi: 10.1186/s12944-019-1036-1 (PMC6489332; doi:10.1186/s12944-019-1036-1)

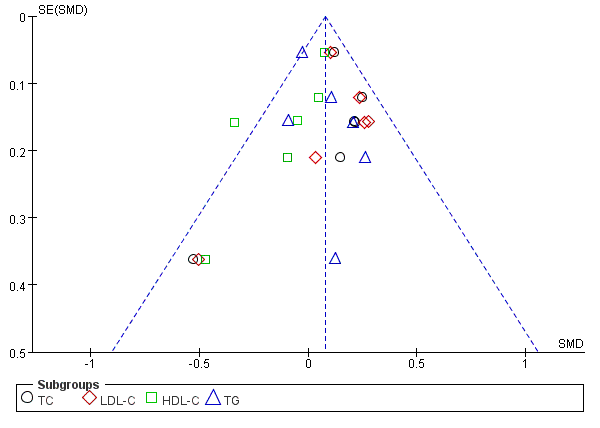

Supplement: Supplementary file 1 — The funnel plot of publication bias detection between PCSK9 rs562556 polymorphism and the serum lipid levels (circle: TC; diamond: LDL-C; square: HDL-C; triangle: TG). (PNG 9 kb) [file 12944_2019_1036_MOESM1_ESM.png]
